# “The Logic of Monsters:” Pere Alberch and the Evolutionary Significance of Experimental Teratology

**DOI:** 10.1007/s10739-024-09783-7

**Published:** 2024-08-30

**Authors:** Juanma Sánchez Arteaga

**Affiliations:** 1 Institute of History (IH), Spanish National Research Council (CSIC), Madrid, Spain; 2 Centro de Ciencias Humanas y Sociales – Consejo Superior de Investigaciones Científicas (CCHS/CSIC), Despacho 2F2, Calle de Albasanz, 26, San Blas-Canillejas, 28037 Madrid, Spain

**Keywords:** Pere Alberch, Teratology, Evolution, Evo-Devo, Evolutionary morphology

## Abstract

This paper offers an historical introduction to Pere Alberch's evolutionary thought and his contributions to Evo-Devo, based on his unique approach to experimental teratology. We will take as our point of reference the teratogenic experiments developed by Alberch and Emily A. Gale during the 1980s, aimed at producing monstrous variants of frogs and salamanders. We will analyze his interpretation of the results of these experiments within the framework of the emergence of evolutionary developmental biology (or “Evo-Devo”). The aim is understand how Alberch interpreted teratological anomalies as highly revealing objects of study for understanding the development of organic form, not only in an ontogenetic sense—throughout embryonic development—but also phylogenetically—throughout the evolution of species. Alberch's interpretation of monsters reflects the influence of a long tradition of non-Darwinian evolutionary thought, which began in the nineteenth century and was continued in the twentieth century by people such as Richard Goldschmidt, Conrad H. Waddington, and Stephen Jay Gould. They all proposed various non-gradualist models of evolution, in which embryonic development played a central role. Following this tradition, Alberch argued that, in order to attain a correct understanding of the role of embryological development in evolution, it was necessary to renounce the gradualist paradigm associated with the Darwinian interpretation of evolution, which understood nature as a continuum. According to Alberch, the study of monstrous abnormalities was of great value in understanding how certain epigenetic restrictions in development could give rise to discontinuities and directionality in morphological transformations throughout evolution.

## Introduction

*The interest in these monsters is that they show how a culture handles the possible and marks its limits. It is a requirement of the human brain to put order in the universe.*

François Jacob (1977)

This paper offers an historical introduction to the teratogenic experiments of the evolutionary biologist Pere Alberch Vié (1954–1998) in the context of the emergence of evolutionary developmental biology (or “Evo-Devo”) in the last quarter of the last century. Alberch was a leading researcher in the field of embryology and theoretical biology, and in the last stage of his career, between 1988 and 1995, he became director of the National Museum of Natural Sciences in Madrid (MNCN-CSIC). In this paper, we will focus on his particular interpretation of evolutionary teratology in the light of the emerging field of Evo-Devo,^1^ which was centered on the understanding of embryonic development (ontogeny) as a basis for understanding the evolutionary development of life and the kinship relationships between different groups of organisms, both present and extinct (phylogeny). Alberch stood out as one of the main promoters of this unique field of studies between the 1970s and 1990s, and was also the first Spanish scientist to make outstanding contributions to this particular branch of evolutionary biology.

Alberch's scientific work took place during a period in which Evo-Devo experienced a great international boom, driven by the interdisciplinary work of numerous researchers from various fields of knowledge.^2^ Among the many outstanding contributions of this first wave of Evo-Devo, it is worth mentioning some scientific works that brought us closer to a more complex understanding of the relationship between phylogenetic processes and embryonic development. Without pretending to be exhaustive, among many possible examples, we could highlight, the important work of the molecular biologist Jacob (1977), winner of the Nobel Prize in 1965, who conceived of evolution as a process of bricolage; the scientific work of the geneticist Lewis (1978); the work of Wieschaus and Nüsslein-Volhard and Wieschaus (1980), themselves Nobel Prize winners in 1995 for understanding the genetic control of embryonic development; the works of the palaeontologist and evolutionary theorist Stephen Jay Gould (1985) on the relationship between phylogeny and ontogeny; the work of the biophysicist Newman and Frisch (1979), on the dynamics of morphological pattern formation processes; the work of the molecular biologists McGinnis et al. (1984) on the functioning of homeotic genes, the understanding of which led to the research of two other great Spanish scientists, the geneticists García-Bellido et al. (1973) and Morata and Lawrence (1975).

This new field of interdisciplinary research was the product of a long and multifaceted history, in which different research programs from different evolutionary and developmental perspectives, such as comparative and experimental embryology, developmental mechanics, molecular and developmental genetics, heterochrony theory, among others, fed contemporary Evo-Devo (Love and Raff 2003). I this paper I will analyze Alberch's distinct contribution to evolutionary teratology and, more specifically, I will focus on the evolutionary interpretation given by Alberch in his teratogenic experiments, aimed at the study of morphogenesis, that is, the origin and development of organic form, based on the controlled experimental generation of teratological forms in amphibians. In what follows, I will begin by presenting a summary of Alberch's scientific biography. I will continue by situating his teratological research in the long tradition of evolutionary thought which, between the nineteenth and twentieth centuries, attached great importance to the scientific study of monstrous forms and gave them various evolutionary interpretations. This is important, not just for a better understanding of Pere Alberch’s work, but also for a richer understanding of the historical development of Evo-Devo, since, as it has been pointed out, many histories of Evo-Devo overlook its deep historical roots. Exposing this richer history is particularly relevant because it serves to offer historical perspectives for contemporary research efforts in evolution and development.^3^ I will then explore Alberch's teratogenic experiments in the context of the theoretical biology of the 1970s and 1980s, summarizing some of their main implications for evolutionary thought in the same period. Finally, I will try to contextualize Pere Alberch's ideas on the evolution of organic form within the framework of a current of thought which was highly critical of the dominant Neo-Darwinian paradigm, characterized by a gradualist, adaptationist and what is now considered a “gene-centric” approach to evolutionary explanations.

## Pere Alberch Vié (1954–1998): A Brief but Intense Scientific Career

Pere Alberch Vié was born in Badalona in 1954, into a well-to-do Catalan family. From an early age he showed a great interest in natural history, which led him to write his first scientific articles on amphibians at the age of 19. His family's social position allowed him to pursue his university studies in the United States, specifically at the University of Kansas, where at the age of 22 he obtained a double degree in philosophy and systematics/ecology (Etxebarria and de la Rosa 2021). As soon as he graduated, he began his doctoral studies in zoology at the prestigious University of California, Berkeley. There he joined the research group of the evolutionary biologist David Wake, who specialized in salamanders and who served as co-director of Alberch’s doctoral research, together with the theoretical biologist George Oster, a specialist in biological mathematics and dynamical systems theory. This interdisciplinary legacy, which brought together evolutionary biology, herpetology and bio-mathematical models, was to prove extremely fruitful for Alberch.

It has been shown by authors such as Smocovitis (1996) that the crucial moment that made possible the unification of evolutionary biology in the mid-twentieth century came with the successful adoption of experimentation in evolutionary practice through the mathematical models, or systems of interacting variables, established first by pioneers such as R. A. Fisher, J. B. S. Haldane and Sewall Wright. The triumph of the so-called “evolutionary synthesis” thus led to “the quantification of evolution - the attachment of number to ‘nature’ - and the growing measurability and testability of natural selection [that] were part of a process that would eventually lead to general support for natural selection as the primary mechanism of evolution” (Smocovitis 1992, p. 20). Alberch's quantitative model, inspired by Stephen Jay Gould’s morphological ideas, were different, however. They moved away from this bio-mathematical paradigm of the Neo-Darwinian synthesis, in an attempt to offer a quantitative model capable of explaining morphological changes in ontogeny and phylogeny independently of natural selection. During this period, Alberch's thinking was in fact, profoundly influenced by the theoretical models developed by Gould and Niles Eldredge, who were keen to transform evolutionary theory in the 1970s.^4^ Their new evolutionary models proposed alternative pathways to the gradualist and selectionist explanations postulated by Neo-Darwinism. Alberch's doctoral research was aimed precisely at providing a mathematical formalization of Gould's ideas on the relationships between ontogeny and phylogeny throughout evolution and on the processes of embryonic heterochrony; he considered both as essential elements for understanding the origin of organic forms. Alberch's research, supported by Wake, Oster and Gould himself, would result in a co-authored scientific article that would soon become a small “classic” of theoretical biology (Alberch et al. 1979). In this paper, Alberch and his mentors proposed a quantitative model capable of explaining the relationship between heterochronic changes in ontogeny with the morphological variation observed throughout phylogeny, advocating a unified vision of embryology and evolutionary biology in the study of the evolution of organic form.

After receiving his PhD, Alberch was hired in 1980 as Assistant Professor at Harvard University, where he also served as Curator of the Herpetological Section of its Museum of Comparative Zoology (Renzi et al. 1999). Just 1 year after joining Harvard, Alberch participated in the famous Dahlem Conference or *Dahlem Konferenzen* on Evolution and Development. He took part in this scientific meeting and gave another influential paper on the evolutionary implications of internal constraints on the development of organic form in embryonic processes (Alberch 1982). This congress, held in May 1981 in Berlin, was attended by the main proponents of Evo-Devo of the period and became a turning point for the further development of this field of study. Alberch remained at Harvard until the late 1980s, during which time he published his major contributions to evolutionary developmental biology. These works gave him considerable academic prestige, which enabled him to be admitted as a member of the editorial boards of important international scientific journals, such as *Trends in Ecology and Evolution, Biodiversity Letters, Journal of Theoretical Biology*, or the *Journal of Evolutionary Biology* (Renzi et al. 1999). Throughout this period, he worked in collaboration with leading theoretical biologists and bio-mathematicians such as Garrett Mitchell Odell and George Frederick Oster, producing important papers^5^ while directing the scientific work of students such as Neil Shubin, and making important contributions to morphological theory from the unifying perspective of Evo-Devo.^6^ In this period, in which he performed his crucial teratogenic experiments, Alberch sought to establish evolutionary explanations for the appearance of certain morphological and phenotypic patterns both in ontogeny and phylogeny. His work was characterized by the proposal of alternative explanations to natural selection as the main evolutionary mechanism, as well as by his opposition both to phyletic gradualism and to the central role given to genes in the Neo-Darwinian synthesis.

Such a brilliant start to his career was not, however, enough to guarantee him a permanent tenured place at Harvard, a university with 50 Nobel Prize winners among its faculty. Perhaps for this reason, when his hopes of consolidating his career at Harvard were frustrated, Alberch returned to Spain in 1989. Back in his native country, he became Director of the National Museum of Natural Sciences (MNCN) in Madrid. He held this post for 6 years, during which time he led a profound renewal and modernization of the Museum (Albaladejo et al. 2021). In 1995, suffering from health problems, he resigned as director of the MNCN to focus on his recovery. Three years later, in 1998, he accepted an invitation to join a new research center in Valencia, the Cavanilles Institute of Biodiversity and Evolutionary Biology (*Instituto Cavanilles de Biodiversidad y Biología Evolutiva*, or the ICBIBE). Excited about the future that awaited him in this new scientific institution, Alberch had expressed his desire to use the new institute as a scientific platform from which he could “communicate his knowledge through advanced courses on evolution” (Renzi et al. 1999, p. 624).

In what was to be the last stage of his career, Alberch had set out to synthesize the latest advances in dynamical systems theory applied to biology and investigated the interfaces of this emerging theory with chaos, self-organization, and complexity theory. He devoted his last studies in theoretical biology to this task, with the intention of publishing his findings in a book entitled *An Introduction to Chaos Theory and Complexity with a Special Emphasis on Biological Sciences*, a work he was tragically unable to complete (Renzi et al. 1999). Sadly, Alberch was also not able to ever join the ICBIBE, the new Valencian Scientific Institute; he died on the 13th of March 1998 at the age of 43, and while still living in Madrid.

## The “Theoretical Ancestors” of Alberch's Monsters: An Interdisciplinary Intellectual Lineage of Non-Darwinian Evolutionary Thought

Although Alberch's scientific interest was initially in zoology, more specifically in herpetology, the Catalan biologist also wrote on many other aspects of embryonic development and the evolution of form in vertebrates (Alberch 1980, 1982, 1989a, 1991; Alberch et al. 1979; Odell et al. 1981; Oster and Alberch 1982; Alberch and Gale 1983, 1985; Shubin and Alberch 1986). In this sense, Alberch showed broad intellectual concerns, which transcended the narrow framework of empirical science and responded to a genuine interdisciplinary interest in diverse fields of knowledge. In addition to being a prestigious scientist, Alberch was also a fine music aficionado, as well as a notable collector, critic and appreciator of contemporary art. According to some of his biographers, he was even partially inspired by art to devise some of his theoretical models on biological form (Renzi et al. 1999). He also had a keen interest in the humanities, in particular philosophy and history of biology. Alberch's interest in the history of biology, and his interpretation of that history, would undoubtedly merit a separate article. Suffice it to point out here that Alberch's research was crucially marked from his arrival at Harvard by the ideas of development and evolution that Stephen Jay Gould had set out some years earlier in his book *Ontogeny and Phylogeny* (1977), which was largely based on Gould’s distinct historical reconstruction of the ideas of recapitulation, progenesis and neoteny in the history of biology. Alberch himself made use of numerous references to historical sources to illustrate his views on evolutionary morphology. He inscribed his own research on organic form in the wake of a scientific tradition which he himself traced back to the romantic and transcendental ideas of Goethe, Geoffroy St. Hilaire and Owen (Alberch 1989b) and which, not entirely satisfied with the *structuralist* label assigned to this school by Gould, Alberch labelled as the *internalist* tradition. In Alberch’s own words:

I refer to this philosophical tradition as internalist. This approach, instead of focusing on the external forces of evolution, emphasizes the role of internal factors. Internalist theories have a long, but minority, tradition in evolutionary biology […]. Gould (1986) has recently referred to this position as ‘structuralist’ in reference to the theories of Geoffroy St. Hilaire. Given the recent, more restricted usage of the term in anthropology, linguistics and even evolutionary biology (e.g., Lauder 1981; Webster & Goodwin 1982), I prefer to use the more descriptive, and philosophically less loaded, term of ‘internalist.’ The roots of the internalist perspective predate Darwinism and they can be found in the ideas of Romantic and transcendental morphologists such as Goethe, Geoffroy St. Hilaire and Owen, to name only three influential figures from three different countries. These authors, and many others (see historical reviews by Russel 1916 and Appel 1987), searched for invariances and universal rules of organization in support of their basic tenet which argues that form is primary over function and that a ‘unity of plan’ underlies the diversity observed in nature. (Alberch 1989b)

**Fig. 1:**
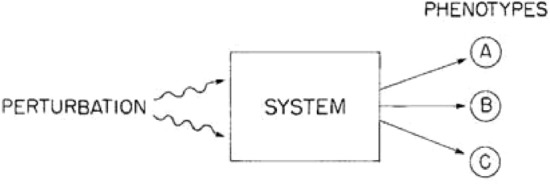
Internalist scheme. Perturbations resulting from genetic mutation or environmental impact are “filtered” through the dynamics of a pattern-generating system. The internal structure of the developmental system defines a finite and discrete set of possible outcomes or phenotypes, even if the sources of perturbation are random. Originally reproduced in Alberch (1989b)

Alberch’s interest in philosophy and history of biology should not be surprising, if we remember that he had graduated from the University of Kansas with a double degree in systematics/ecology and philosophy. In this sense, his theoretical perspective reflected the heritage of a tradition of “internalist” or “structuralist” evolutionary thought, according to which internal structural factors, of a physicochemical and mathematical order, carried more weight than natural selection in understanding the origin of the organic form. His perspective was based on bio-mathematics, an essentially interdisciplinary field of study, inaugurated by D'Arcy Wentworth Thompson in his famous 1917 book *On Growth and Form*, which, incidentally, was also read widely and inspired artistic movements. Gould, who became a mentor to Alberch, summarized this bio-mathematical perspective as a hybrid theory based on Pythagoras and Newton, which argued that physical forces shape organisms directly and that the “internal” and genetic forces are only responsible for producing the raw materials and the form of a construction under the principles of physics (Gould 2011). In line with this approach, Alberch oriented his gaze towards teratology from an epistemological position that could be defined as an *internalist structuralism*, which he conceived as a new research program in evolutionary biology, more focused on the internal properties of the system than on adaptation (Alberch 1989a) (Fig. 1).

Alberch's view of organic transformations and the origin of biological form gave a central role to the physicochemical laws governing the process of embryonic development. In this sense, we can include Alberch among the most prominent advocates, at the end of the last century, of what the philosopher and historian of science Ron Amudson calls the developmentalist doctrine on the explanation of evolutionary change:

The developmentalist doctrine conceives of evolutionary change between species as a change in the embryological processes that give rise to individual morphologies. To understand evolutionary change, one must first understand the processes of individual development, the ontogenetic processes that produce adult morphologies from single cells. These processes vary between species in a particular lawlike way, described by von Baer. They vary so as to produce adult morphologies whose taxonomic relations are expressed by the hierarchical Natural System. The evolution of one species into another species can only happen as a result of changes in the ontogenetic processes by which the species’ morphology is constructed. If the ontogenetic processes don’t change, the adult morphology cannot change. If our study of embryology and comparative morphology is successful, it may allow us to understand not only how existing morphologies are generated by related ontogenetic processes, but also how ontogenetic processes can change, resulting in new forms and new species. (Amundson 2005, p. 90)

This tradition of scientific thought, which Amundson calls “developmentalist” converges in Alberch’s interpretation of evolutionary biology with another current of biological thought that authors such as Gerry Webster, Brian Goodwin, or Gould himself labelled as “structuralist,” and which Alberch himself referred to as “internalist.” In Alberch's time, it was basically postulated as a criticism of the primacy of natural selection as the causal agent of organic form, which was instead explained by epigenetic factors, internal to the embryonic development itself. In the words of the Webster and Goodwin (1982), the structuralist critique of the Neo-Darwinian Synthesis was

conducted entirely in terms of the problem of biological form—how to account for the existence of a diversity of specific morphologies which can be classified in some hierarchical manner—since this is an area […] distinguished by the absence of an adequate theory of the production and reproduction of these forms. We do not believe that this failure to produce a satisfactory theory is a consequence of the supposed difficulty of the problem but, rather, that it is a consequence of the intrinsic inadequacy of the current system of concepts. It is our contention that without a change in the system, no progress can be expected in this crucial area. We suggest that a more satisfactory conceptual system involving a structuralist conception of the organism can be developed from seeds found in the tradition of Cuvierian rational morphology and in the work of a number of fringe figures somewhat outside the mainstream of twentieth-century biology who, consciously or unconsciously, have allied themselves with this tradition. (Webster and Goodwin 1982, pp. 38–39)

For Alberch, the constraints imposed by the mathematics underlying the physicochemical processes that take place during embryonic development were far superior to natural selection in explaining the origin and evolution of organic form. While it was undeniable that, along the phylogeny, natural selection acted on genes to “filter” the forms best suited to the environmental conditions of each species, Alberch was at pains to convey the idea that natural selection could only act *a posteriori* on a set of genes previously “filtered” and adjusted to the set of possible forms for the embryo. This set of possible forms—called the phenotypic space, or morphospace—was limited *a priori* by the physicochemical conditions of the dynamic system of the organism, which only allowed for a finite set of structurally viable phenotypes.

**Fig. 2:**
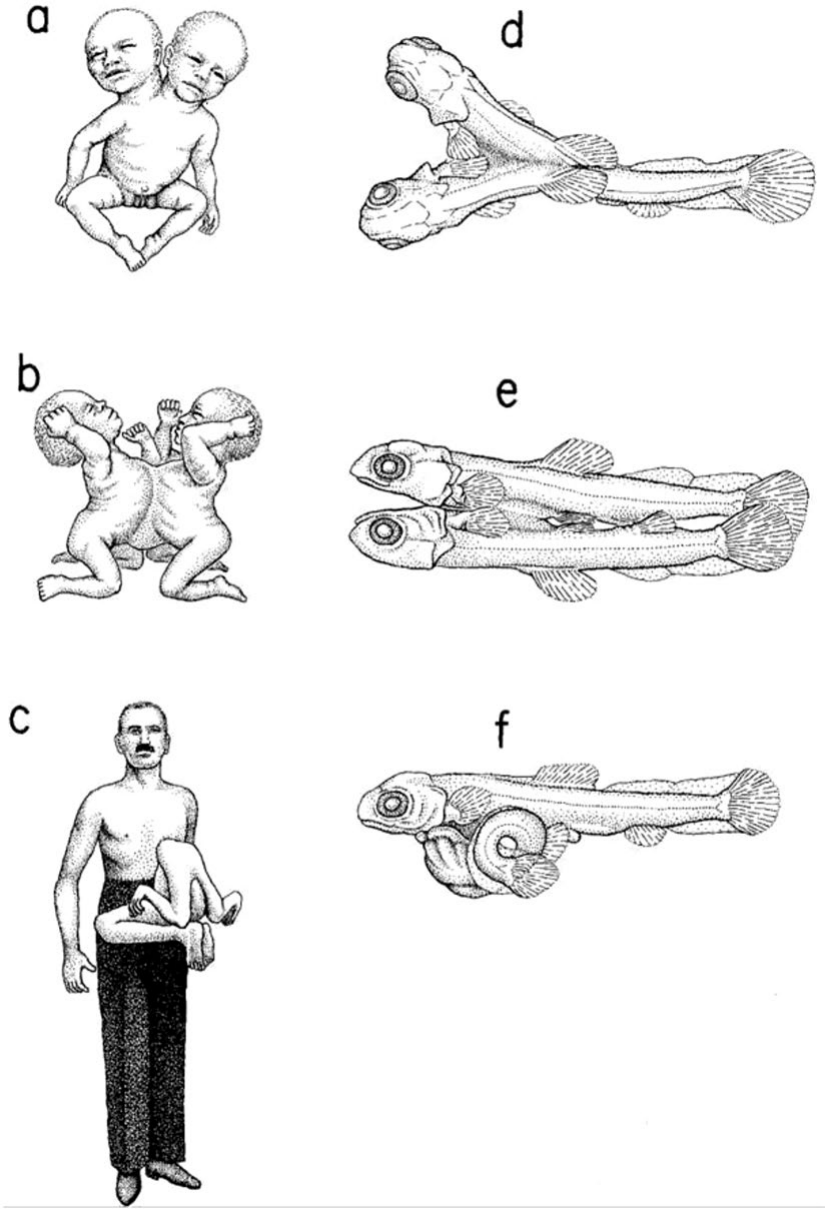
Ordered variation and trans-specific parallelism in the occurrence of monstrosities. The three basic types of conjoined twins (“double monstrosities”) in humans (**a**–**c**) and in the fish *Fundulus* (**d**–**f**). Duplication is the result of anterior bifurcation of the body axis (**a**, **d**). Fully developed twins can “fuse” through any region of the body, but most often fusion occurs in the abdominal region (**b**, **e**). Parasitic twins are also observed in all vertebrate taxa (e.g., **c**, **f**). Originally reproduced in Alberch (1989b)

Monsters, or teratological variants of living organisms, were particularly well suited to understand these internal morphological constraints, independent of adaptive factors, since, being unviable creatures under natural conditions, natural selection could not be invoked to explain their capricious morphologies. Considering these internal constraints on development, Alberch argued that the improbability and low statistical significance of certain teratological morphologies, such as three-headed monsters, could be explained without any reference to natural selection. By the same kind of reasoning, it would be easy to explain why some frequent teratological morphologies, such as cases of two-headed monsters in various vertebrate groups were common. Because of these internal structural factors of embryonic development, the regularity observed in certain teratological morphologies present in very diverse species could be understood, without recourse to natural selection (Fig. 2).

Going beyond the general bio-mathematical framework inaugurated by Thompson (1917) as the basis for understanding the origin of organic form, there was another British biologist of the twentieth century whose influence was, if possible, even more important than Thompson's for understanding Alberch's work in the field of quantitative morphology. I am referring to the embryologist and geneticist Conrad Hal Waddington. In fact, much of the theoretical backdrop that underlies the bio-mathematics proposed by Alberch and his collaborators in explaining morphogenesis was directly inspired by Waddington's 1957 book, entitled *The Strategy of the Genes.*^7^ In this book, conceived independent of the orthodox stream of the Neo-Darwinian mathematical population geneticists, such as R. A. Fisher, J. B. S. Haldane or Sewall Wright, whose work he respected, but considered irrelevant, Waddington (1957) demonstrated enormous originality, and conceived of embryonic development as a process of morphological transformation unfolding along what he termed the “epigenetic landscape.” Morphological development could be understood with the metaphor of a pinball machine, in which the ball moved primarily down the main valley to produce normal phenotypes, but occasionally drifted into a secondary valley and produced a mutant phenotype (Robertson 1977). In other words, the development of embryonic form could be understood as the trajectory of a ball rolling down a hillside, whose contours, or *chreods*, in Waddingtonian terminology, would channel the ball's progress and the development of form in a certain direction, hindering its progress in other directions. In the case of a deeply dug channel or chreod in the slope of the “epigenetic landscape,” it would be highly unlikely that any external perturbation, for example a selective pressure caused by certain environmental changes, would succeed in modifying or preventing the normal morphological development. Thus, the morphology of the organism would be “channeled” *a priori* by the structure of the epigenetic landscape. Thus, the final shape of the organism would depend not only on its genetic make-up, but also on the different ways and degrees to which genes could be expressed throughout embryonic development in different regions of the epigenetic landscape. Waddington (1953) created the first counter-synthesis argument, calling for further synthesis between embryology and evolutionary biology (Smocovitis 1996). His ideas were highly influential to subsequent embryologists and developmental biologists, such as Alberch. Waddington’s criticisms of Neo-Darwinian evolutionary theory were focused on what Adam Wilkins, biological theorist and historian, described as “unrealistic, atomistic models of both gene selection and trait evolution. In particular, he felt that the Neo-Darwinians badly neglected the phenomenon of extensive gene interactions and that the randomness of mutational effects, posited in the theory, was a false postulate. This last criticism dealt with the phenomenon known today as developmental constraints” (Wilkins 2008, p. 224). Clearly influenced by Waddington’s thought, the bio-mathematical work of Alberch and his collaborators in the field of theoretical morphology was based on the hypothesis that the various possible ontogenetic trajectories could be quantified mathematically, transforming the Waddingtonian chreods into a set of differential equations capable of expressing the morphological transitions likely to be experienced by the embryo (Alberch et al. 1979).

This interest in studying morphological transformation based on quantitative analysis of multiple parameters was set early in Alberch's scientific career as a researcher. Prior to his teratogenic experiments, the mathematical expression of morphological transformation processes in ontogeny and phylogeny was already present in Alberch's first relevant contribution to theoretical biology. I am referring to the already mentioned article that, at the age of just 25, Alberch signed as first author together with Gould, Oster, and David Wake, and that would soon become an obligatory reference for any student of Evo-Devo (Alberch et al. 1979). In this work, entitled “size and shape in ontogeny and phylogeny” Alberch and his collaborators proposed a mathematical model consistent with the theoretical framework that Gould had previously set out in his 1977 book *Ontogeny and Phylogeny* to explain heterochrony, the relative changes in ontogenetic development times between different species. More specifically, the quantitative model designed by Alberch and his colleagues was intended to describe the various types of heterochronies described by Gould and the relationship between these different heterochronic changes and the phyletic trends observed in phylogeny (Nuño de la Rosa 2016). A few years earlier, Gould had theoretically postulated that alterations in the rhythm of embryonic development could give rise to two types of parallelism between ontogeny and phylogeny. First, a direct parallelism (or recapitulation) could occur when a trait of the descendant species appeared at an earlier stage of ontogeny than that of the ancestral species, by a heterochronic process called neoteny. But an inverse parallelism between ontogeny and phylogeny could also occur by a heterochronic process called paedomorphosis when the trait appeared relatively later in the ontogenetic development of the descendant species than that of the ancestor. Accepting this theoretical framework, Alberch and his eminent colleagues, including Gould, postulated a growth law for organic size and shape that could be expressed as a mathematical function (Y) from a set of differential equations. The model considered as independent variables the age of onset of growth, (a); the age at which the structure under study completed its differentiation and growth, (b); the growth rate, (k) and the initial size of the organism (Sº) at the age (a) of the system. According to this model, both the morphological evolution along the phylogeny and the ontogenetic development of organisms could be expressed as the product of the temporal modification of these parameters in the ontogenetic trajectories of the ancestor and descendant species. Based on these assumptions, a metamorphic process could be expressed mathematically as a function dependent on quantitative modifications between these parameters (Renzi et al. 1999).

In addition to this predilection for bio-mathematics and Waddington's embryological theories, Alberch's teratogenic experiments also demonstrated a remarkable interest in the history of experimental teratology. Alberch was aware that his scientific work on monsters (*terata*) was heir to a long historical tradition focused on the study of biological “monstrosity” as a source for understanding the origin and development of organic form throughout the history of species (Alberch 1989a, 1989b). In this sense, likely inspired by Gould, his admired mentor, Alberch's interest in the history of science led him to situate his own teratological research in the wake of a long theoretical–experimental tradition of non-Darwinian evolutionary thought, which the Neo-Darwinian synthesis seemed to have left aside. In this sense, Alberch's teratogenic experiments can be linked to a pre-Darwinian evolutionary tradition,^8^ which initially arose in the context of the transformist thinking inaugurated at the beginning of the nineteenth century by Jean-Baptiste Lamarck. This intellectual tradition was far removed from the Neo-Darwinian paradigm that gave a central role in evolution to the gradual adaptation of organisms to their environment because of natural selection.

Long before Darwin began to speculate on the origin of species by means of natural selection, the eighteenth century Frenchman Étienne Geoffroy de Saint-Hilaire, a fellow countryman and direct collaborator of Lamarck, was the first to point out that the controlled production of monstrous embryos of different species was an ideal experimental method for testing some of the great hypotheses put forward by the theory of the transmutation of species, i.e., what eventually was called evolution (Fischer 1972). The methodology of experimental teratogenesis began to be systematized in the first half of the nineteenth century by Geoffroy de Saint-Hilaire (1822). He was also a pioneer scientist in the field of evolutionary morphology to whom some historians have recently attributed the role of pioneering *avant la lettre* of Evo-Devo (Panchen 2001), and even of the epigenetic theory of evolution (Iurato and Igamberdiev 2021). For Saint-Hilaire, monsters played a key role in understanding the emergence of new organic forms and were essential for understanding the evolutionary emergence of new groups of organisms. Based on these premises, he even went on to postulate the first known embryological model to explain the evolution of vertebrate animals (Galera 2021). Applying the environmentalist reasons of his mentor Lamarck, guided by the results obtained in his laboratory, Étienne Geoffroy de Saint-Hilaire articulated an unprecedented transformist theory based on teratology as the general cause of the phenomenon. The genesis of a new species would occur in the embryonic stage through the formation of monsters: units catalogued as products contrary to the normal reproductive order. Expressed in such terms, morphogenesis constituted the operative mechanism of evolution, and organic transformation meant the organizational modification of living matter (Galera 2015). During the rest of the nineteenth century, and following the path opened by Étienne Geoffroy Saint-Hilaire, experimental teratology was further developed and sophisticated by his son Geoffroy de Saint-Hilaire (1836) and by Dareste (1877, 1891). The latter went a step further, systematizing the basis of what he called *teratogeny*, i.e., the experimental production of monsters for the study of evolution. As noted by the French philosopher and physician Georges Canguilhem:

Dareste proclaimed that teratogeny should create its object [...] He flattered himself that he had succeeded in producing simple monstrosities in chicken embryos, according to the classification of Isidore Geoffroy Saint-Hilaire, and he hoped to succeed in producing hereditary varieties. Encouraged by Darwin’s appreciation of his experiments (‘full of promise for the future’), Dareste hoped to use the resources of experimentation to elucidate the origin of species. (Canguilhem and Jaeger 1962, p. 38)

The tradition that united embryology and experimental teratology with what today we would call evolutionary biology, inaugurated by Saint-Hilaire and Dareste, was taken up again in the twentieth century. Monsters continued to be considered as particularly significant organisms for understanding evolution by important heterodox evolutionists, or at least “marginal” in relation to the “hard core” of the so-called Neo-Darwinian theory. Scientists such as the German physiological geneticist Richard Goldschmidt or Waddington reinforced the theoretical interest of teratology for evolutionary biology (Diogo et al. 2017). The common thread in all these scientists, and what makes it possible to speak of a certain “intellectual tradition,” which would have a decisive influence on Pere Alberch's scientific work, is that they all proposed evolutionary models in which a central role was given to ontogenetic development and to the structural internal restrictions that determine the evolution of the embryo's form as keys to understanding morphogenesis. Moreover, all of them considered these “internal” ontogenetic factors as primordial in understanding the origin of organic form, placing them far above the action of “external” or adaptive factors, such as the action of genes subject to natural selection (Galera 2015). Moreover, in line with the punctuated equilibrium theory advocated in the 1970s by Eldredge and Gould, some of these models provided plausible hypotheses to explain abrupt morphological transitions in phylogeny (Galera 2021). This was a phenomenon repeatedly observed in the fossil record, for which the Neo-Darwinian Synthesis with its gradualist, selectionist and “gene-centric” approaches that dominated evolutionary biology in Alberch’s time, did not offer a satisfactory answer.

## The Evolutionary Significance of Alberch's Teratogenic Experiments

In a bibliographical review published in 1983 in the journal *Science*, Alberch commented on the recent work of the biologists Rudolph Raff and Thomas Kaufmann on the relationship between embryology and evolution.^9^ There, the Spanish biologist summarized some of his main ideas regarding the direct link between evolutionary morphology and the study of embryonic development. According to him, in the light of recent findings in the field of Evo-Devo, the biological organism could no longer be seen as a kind of “black box” in which we ignore all the internal mechanisms, and whose morphology could only be explained as an adaptive response to the environment by the effect of natural selection. Contrary to these ideas, Alberch upheld the view that recent research in evolutionary developmental biology suggested that morphological evolution was discontinuous. However, far from any dogmatism, he recognized that both the role of natural selection in the origin of organic forms and the cause of the morphological discontinuities observed in phylogeny were open questions, the elucidation of which would clarify basic properties of evolutionary processes (Alberch 1983). Alberch's teratogenic experiments made sense precisely because they attempted to answer these two questions, which were considered deeply controversial in evolutionary biology at the time. In the remainder of this section, I will try to synthesize Alberch's distinct and unique conception of experimental teratology as a valuable source of data for understanding the relationships between embryology, evolution, and morphogenesis. Finally, I will try to contextualize his main contributions to evolutionary morphology within the framework of Evo-Devo in the last quarter of the twentieth century.

During his early career, Alberch, in collaboration with Emily A. Gale, worked on analyzing the teratological forms generated experimentally in amphibian embryos by the controlled administration of certain substances that caused malformations during tadpole development (Alberch and Gale 1983, 1985). In a paper given at the International Colloquium on Ontogenesis and Evolution held in Dijon in 1986, Alberch addressed this issue. There, he argued for approaching evolutionary problems related to organic morphogenesis by the careful study of artificially generated deformations. Based on these premises, Alberch and Gale set out to produce deformations in the limbs of frog and salamander embryos to study the morphological significance of their *terata* in evolutionary terms. Their teratological variants were examples of organic morphologies with no adaptive value, and were therefore, independent of natural selection, but nevertheless were perfectly viable as organisms from a structural point of view. Taking up the old experimental tradition of evolutionary teratology that linked Étienne Geoffroy de Saint-Hilaire with Waddington, Alberch argued that experimentally induced teratological anomalies in the embryonic development of his experimental monsters could reveal important clues to understanding the origin and development of limb form in the phylogeny of these amphibians and, by extension, in the evolution of all vertebrates.

Alberch and Gale examined the effects of colchicine—a mitotic inhibitor that causes limb shrinkage and loss of skeletal elements—on the fingertips of the frog *Xenopus laevis* and the salamander *Ambystoma mexicanum*. Alberch and Gale's experimental work was supported by a series of earlier experiments conducted in the middle of the century by Swiss biologists Alfred Bretscher and Pierre-André Tschumi.^10^ Bretscher had reported a series of experiments demonstrating that local treatment of the embryonic bud of the frog *X. laevis* with the mitotic inhibitor colchicine resulted in the reduction, and even loss, of toes (Bretscher 1949). These experiments were further elaborated and discussed in Bretscher and Tschumi (1951) and Tschumi (1954). Based on this information, the Swiss researchers pointed out some of the possible evolutionary implications of their results and drew an analogy between the patterns of experimental digital reduction and loss and the phyletic tendency to limb reduction observed in some groups of reptiles and mammals. Subsequently, Rensch (1959), Waddington (1962), and Devillers (1965) reported Tschumi's results and their possible implications for evolutionary studies. These experiments also raised some interesting questions about development, in particular, the relationship between size and pattern formation in embryonic processes. Bretscher and Tschumi imagined that the development of amphibian limbs depended on the size (roughly equivalent to the number of undifferentiated cells) of the embryonic limb bud, a hypothesis later corroborated by Alberch and Gale (1983).

In fact, Alberch and Gale set out to perform a series of experiments in which they modified and extended the experimental protocol of Bretscher and Tschumi. To do so, they locally treated with colchicine the limb buds of the anuran *X. laevis*, and the salamander *A. mexicanum*, throughout various stages of limb development. Unlike Bretscher and Tschumi, however, Alberch and Gale focused on the differentiation of individual skeletal elements along the ontogenetic development, and not on measures of external size. Furthermore, unlike Bretscher and Tschumi, Alberch and Gale were interested in measuring specific developmental stages at different times of treatment, and quantified differences in growth rates between experimental and control limbs. In their experiments, both *Ambystoma* axolotls and *Xenopus* frogs were treated using a slightly modified method adapted from Bretscher (1949). The amphibians’ larvae were anesthetized in a 1:7000 solution of ethyl *m*-aminobenzoate and placed, left side up, in a small cavity carved in a paraffin-filled Petri dish. The head and tail of the animals to be treated were covered with moist paper towels to prevent desiccation (but not so much as to cause suffocation). Under a dissecting microscope, they punctured the left limb at four evenly distributed points with a sharp tungsten needle, a necessary procedure to aid rapid diffusion of colchicine into the limb bud. After that, they placed a small piece of tin foil under the limb bud, isolating it from the underlying body wall. They then covered the bud with filter paper (approximately 1–5 mm) saturated with a 1:2000 solution of colchicine. The larvae were treated for 25 min. After that period, the filter paper and tin foil were removed. Since the right foot was not affected and developed normally, it was used as a control measure for each animal. In addition, the same experimental manipulations were repeated on the control limb buds, except that the filter paper was soaked in Holtfreter's solution instead of colchicine. Most of the animals—more than 90%—survived this treatment.

To quantify differences in growth rates between experimental and control limbs, the contours of both feet of the treated larvae of both species were drawn at the time of treatment. This was repeated every 5 to 7 days for the next 9 weeks. The area of each foot was calculated each week using a digitizer. Individual longitudinal growth curves were obtained in this way, being the only quantitative analysis that was derived from these experiments. Ontogenetic series of both species were prepared by retaining groups of three salamanders at 5-day intervals and five *Xenopus* at 7-day intervals during hindlimb development.

The results of these experiments showed two types of foot reduction in both *Xenopus* and *Ambystoma*: colchicine treatment could result in loss of an entire digit or loss of individual elements (phalanges). Both *Xenopus* and *Ambystoma* showed digit loss in approximately one-third of the cases. However, in *Xenopus*, digit 1 (the “thumb”) was lost much more frequently than any other digit. In contrast, axolotls of the genus *Ambystoma* only lost digit 5. In addition, the loss of individual phalanges also showed a different pattern in *Xenopus* and *Ambystoma*. Alberch and Gale's results also suggested that the shape of the amphibian limb depended on the number of undifferentiated cells or the absolute size of the limb bud, and that the mechanisms of digital differentiation along the anteroposterior and proximodistal axes were, to some extent, independent, i.e., that the loss of an entire digit was not necessarily accompanied by extreme proximodigital reduction of the remaining elements.

The evolutionary implications of these results were of great interest. Alberch argued that the teratological variants generated experimentally in his experiments were particularly illuminating for the role of natural selection in morphogenesis, as well as for morphological discontinuity in evolution. If amphibian morphology depended exclusively on natural selection, one would expect that teratogenic agents fed to frog and salamander embryos would generate an unrestricted variability of unviable monstrous forms. That is, if selection were the primary determinant of morphology, operating by selecting viable phenotypes and eliminating the rest, one would expect the chemical agent fed to tadpoles to cause a random variety of morphological alterations in amphibian limbs. According to the selectionist hypothesis explaining the origin of organic forms, this initial morphological diversity of the experimental *terata* would be limited *a posteriori* by natural selection, which would eliminate the least fit forms and allow only the morphologies most suitable for survival to reproduce.

**Fig. 3:**
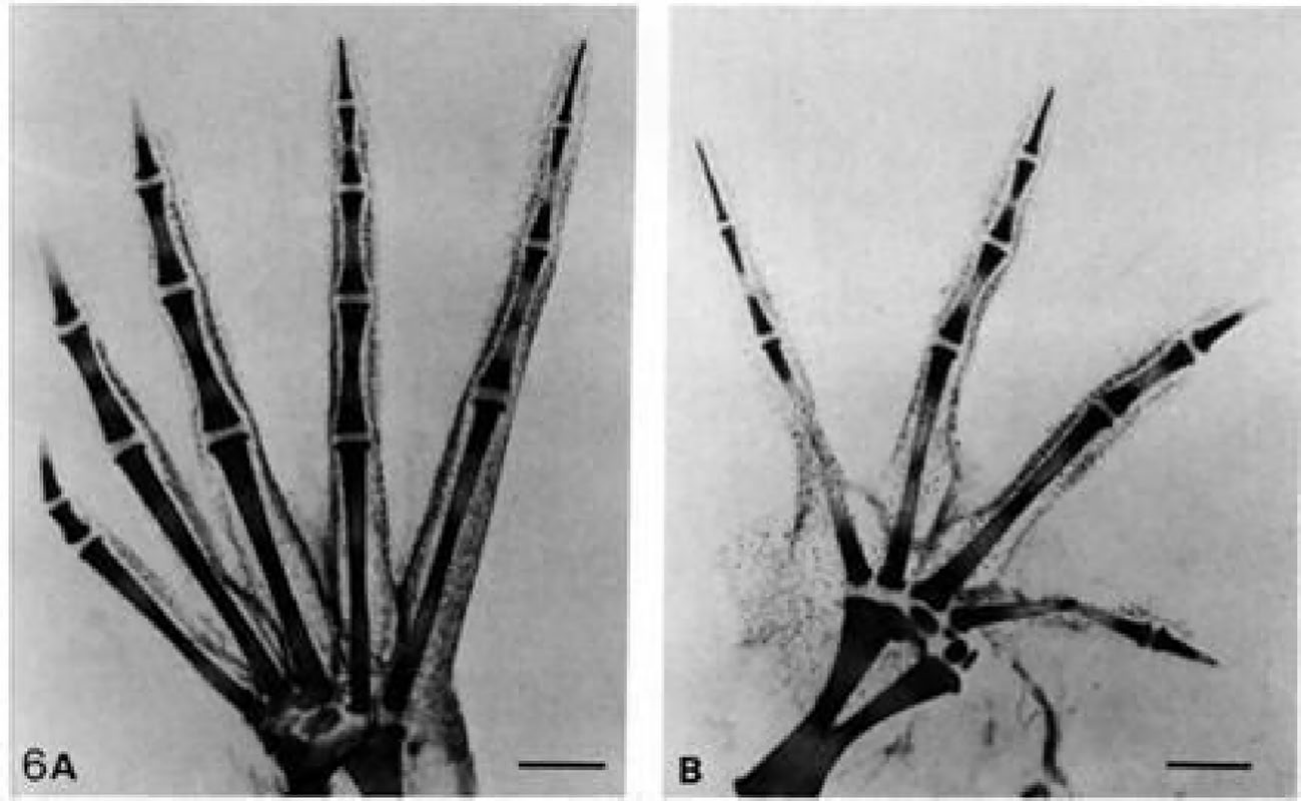
Teratogenic effects of colchicine on the limbs of the frog *Xenopus laevis*. Rinsed and stained control right foot (**a**) and treated left foot (**b**) of *Xenopus laevis*. Note the smaller size of the treated foot. Originally reproduced in Alberch and Gale (1983)

However, the *terata* produced experimentally by Alberch and Gale did not follow this adaptationist model. The experimental monsters exhibited a limited number of patterns of variation, and these patterns were not “random,” but were clearly groupable into a restricted set of typologies, just as they were in nature among “normal” individuals (Renzi et al. 1999). Furthermore, Alberch and Gale observed that the experimental monstrous varieties responded to a clearly differentiated pattern in each group, with clear differences between anurans (frogs) and urodeles (salamanders): frogs that had lost a toe always lose the first toe, while salamanders always lose the fifth (Alberch and Gale 1983). Surprisingly, these same artificially generated patterns of morphological differentiation among the *terata* corresponded to the differences observed in nature in other frog and salamander species, related to the species they had taken as experimental models. According to Alberch, the experimental perturbations produced in his *terata* generated results that paralleled the ontogenetic and phylogenetic differences observable in nature (Alberch and Blanco 1996). In other words, when studying *in natura* the embryonic developmental sequence of digital differentiation of several species of frogs close to *X. laevis*, different stages of development of the first finger were observed, distinguishing typologies very close to those of frog embryos modified in the laboratory. In other words, the natural morphological variability in these anuran species showed the same pattern observed when colchicine was applied to *X. laevis* tadpoles (Fig. 3). In a similar way, salamander species could also be found in the wild with loss or malformation of the fifth toe, as was the case with the tadpoles of *A. mexicanum* to which Alberch had given colchicine.

**Fig. 4:**
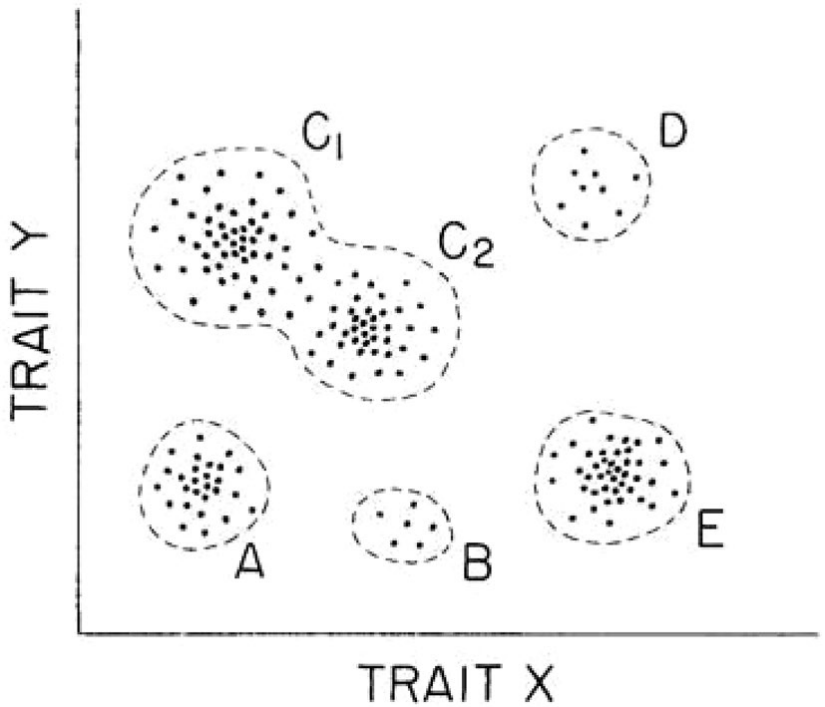
A phenotypic space is the set of all possible phenotypes. This diagram assumes that the morphology of an organism can be completely defined by measuring two traits, x and y. Each point corresponds to a specific morphology. Not all possible phenotypes are found. The distribution of points is not uniform. Groups of points may correspond to species (e.g. A and E), to polymorphic or closely related species (e.g. C1 and C2) that are not separated by distinct morphological discontinuities, or to classes of teratologies (e.g. D and B). Originally reproduced in Alberch (1989b)

For Alberch, this surprising experimental result had profound implications for understanding the relationships between ontogeny, phylogeny, and morphogenesis outside the Neo-Darwinian interpretative framework. First, Alberch and Gale's experimental results seemed to show that the morphospace was discontinuous in the case of these amphibians. Only a limited number of phenotypes existed among the colchicine-modified variants. In Alberch's words: “not all possible phenotypes were found. The distribution of points [in the morphospace] was not uniform” (Alberch 1989b) (Fig. 4).^11^

The interaction between the teratogenic environment and the colchicine-treated organisms produced a finite set of phenotypes out of all conceivable forms: colchicine administration generated only a limited subset of phenotypes in the embryonic development of the experimental monsters. On the other hand, the regularities and discontinuities observed in the morphologies of colchicine-treated tadpoles could not be explained by the effect of natural selection. For Alberch, the results of his teratogenic experiments were compatible with a view of evolution in which natural selection would operate only as an agent of secondary morphological constraint, as opposed to the primary morphological constraint established *a priori* by the internal physicochemical laws of embryonic development for these organisms (Oster and Alberch 1982). From this perspective, the Neo-Darwinian synthesis could only predict which morphologies would survive in each environment, but not which phenotypes would most likely emerge during ontogenetic development, prior to the action of natural selection (Alberch 1982). Thus, Alberch and Gale's experimental results seemed incompatible with the selectionist, gradualist and gene-centric assumptions associated with Neo-Darwinian synthetic theory, which understood nature as a continuum shaped by natural selection. Away from this interpretative paradigm, Alberch argued that the morphological discontinuities observed among his experimental *terata* responded to an internal structural order, unrelated to selective pressures. The internal structure of the developmental system defined a finite and discrete set of possible outcomes (phenotypes), even if the sources of perturbation were random (Alberch 1989a, 1989b). Epigenetic structural constraints operating in the embryonic system of frog and salamander tadpoles acted as a hierarchical causal agent, superior to gene action and natural selection in explaining the morphogenesis of experimental monsters. The internal physicochemical limits of the embryonic morphogenetic field restricted *a priori* the set of possible forms on which natural selection could act later, just as a secondary factor of morphological determination.

Thus, we arrive at the concept of *evolutionary constraints to development* (Alberch 1982, 1989b), probably one of Alberch's main contributions to evolutionary morphology. This was a concept showing a clear Waddingtonian influence, which, in the last decades of the twentieth century, served to propose an extension of the paradigm offered by Neo-Darwinian synthetic theory in explaining the evolution of organic form.^12^ In contrast to the “externalist,” “selectionist” and “gene-centric” interpretative framework of Neo-Darwinism, Alberch understood the living organism as a complex entity, endowed with an internal structure and organization that restricted *a priori* the possible responses to selective pressures (Alberch 1983), thus limiting the possible morphologies for each group of organisms, completely independent of natural selection. The perturbations resulting from environmental impact were epigenetically “filtered” through embryonic development, which Alberch interpreted as a dynamic system, generating discrete and discontinuous morphological patterns. Thus, for Alberch, embryonic development acted as both the effect and the cause of gene expression (Renzi et al. 1999). The action of genes and natural selection as causative agents of organic form was subordinated to the internal laws of embryonic development in a double sense. Firstly, the action of the genes responsible for protein synthesis—on which natural selection could act—depended on the regulatory genes of ontogenetic development, responsible for their inactivation or activation at different moments and regions of the embryonic morphogenetic field. Secondly, embryonic morphogenesis was determined by the interaction of various biophysical and biochemical parameters operating during embryonic development. The interaction of these parameters established a complex dynamic system, in which the morphogenetic fields were structured according to different gradients and polarities, amenable to quantification and biomathematical analysis.

In short, Alberch's experimental monsters seemed to demonstrate that the embryonic development of the organism corresponded to a dynamic system, in which small alterations in certain physicochemical parameters of the system could produce important qualitative changes in the morphology of the embryos, while at the same time making the appearance of certain forms difficult or impossible. Thus, confronting the Neo-Darwinism that dominated biology at the end of the last century, Alberch argued that the internal structural factors that regulated embryonic morphogenetic fields could give rise to morphological discontinuities, both in ontogeny and phylogeny, completely independently of selective evolutionary processes.

## Final Remarks

As an evolutionary biologist, Alberch objected to the overemphasis that the prevailing Neo-Darwinism of the late twentieth century had placed on genes and on functionalist and adaptive explanations for interpreting evolutionary change. For Alberch, the origin of organic form could not be explained as the exclusive result of natural selection operating to eliminate less adaptive genetic mutations. The artificial production of teratological varieties, by making it possible to study non-adaptive yet structurally viable organisms, offered an exceptional platform on which to empirically analyze the internal mechanisms governing the emergence of morphological diversity outside the selectionist interpretative framework, both ontogenetically and phylogenetically. The “logic of monsters” originated in the evolutionary constraints on development. These internal constraints of the ontogenetic dynamical system made it possible to understand the origin of the discontinuities, gaps and morphological directionality observable in the evolutionary history of life.

According to Alberch, morphological forms and absences could be expressed mathematically, based on a careful parametric quantification and the appropriate differential equations to describe the possible and impossible metamorphoses within the morphospace assigned to each group of organisms. But despite their undisputed originality, the mathematical models developed by Alberch and his collaborators to explain the processes of morphogenesis in ontogeny and phylogeny eventually lost scientific influence. Among the causes of this decline we can perhaps point to the irreducible complexity of biological systems to any one “logical system,” as well as to certain internal errors in the theory of punctuated equilibrium that supported such mathematical models. As Gould himself acknowledged, two basic principles of punctuated equilibrium affecting those models were shown to be wrong: the direct acceleration of the evolutionary rate by the process of speciation (cladogenesis) and the idea that stasis is due to internal resistance to natural selection (Futuyma 2002). But, above all, we can attribute that decline to the new and very powerful tools provided by emergent molecular genetics applied to embryonic development. Despite all this, and despite the opinion of biologists such as Jean Rostand, who in the mid-twentieth century had predicted that experimental teratogenesis would never shed any light on the processes of evolution (Rostand 1964), Alberch's experimental work in the field of teratology continues to be a fertile theoretical framework for the study of evolutionary morphology today (Diogo et al. 2017).

In 1995, Pere Alberch left his position directing the MNCN in Madrid. In 1997, just as he was planning to move to Valencia to start a new research program, he died at the early age of 43. A heterodox scientist with a great innovative capacity, Pere Alberch was not only the first prominent Spanish researcher in the field of Evo-Devo (Baguñà, 2009), but also one of the great international promoters and articulators of the joint study of embryonic development and evolution in the last quarter of the twentieth century. His peculiar interpretation of “the logic of monsters” is a good example of the originality and scientific relevance of his work for the history of evolutionary biology.

## Footnotes

1 Guinard (2015, 2020) defines evolutionary teratology as an interdisciplinary field of research, originated in Étienne Geoffroy Saint-Hilaire’s work, whose basic principle is that evolution of life is marked by the production and sustainability of anatomical structures of teratological nature. It is important to note that this name was not used by Saint-Hilaire or Alberch.

2 See Amundson (2005) and Hall (2012) for more on the historical backdrop to the rise of Evo-Devo.

3 See Love and Raff (2003) for more discussion of how most histories of Evo-Devo have underestimated the deep historical roots of the field, which they also argue is important for gaining perspective on contemporary research in evolution and development.

4 See Eldredge and Gould (1972), Gould (1977), Gould and Eldredge (1977) and Gould and Lewontin (1979) for some of the work calling for reform of evolutionary theory.

5 See Odell et al. (1981) and Oster and Alberch (1982).

6 See especially the paper by Shubin and Alberch (1986).Tschumi

7 Like Alberch, Waddington also had a deep interest in art and even wrote a book dedicated to analyzing the relationship between art and the natural sciences in the twentieth century (Waddington 1969). I will leave open the hypothesis that this lesser known facet of Waddington also contributed to Alberch's fondness for contemporary art.

8 See especially Bowler (1992).

9 See Raff and Kaufmann (1991) for the original work.

10 A detailed description of the experimental procedures used by Alberch and Gale can be found in Alberch and Gale (1983).

11 Morphological discontinuities can be represented by studying the morphospace as the set of all possible phenotypes for a given taxon. In mathematical terms, we can represent morphospace by assuming that the morphology of an organism can be defined by a set of parameters. In the simplest model, we could assume that the shape of an organism would depend on the combination of two traits, X and Y, with each coordinate point (x, y) corresponding to a specific morphology associated with a position in morphospace.

12 The relationship between morphology and Neo-Darwinism has received insufficient attention by historians of biology. One notable exception is Waisbren (1988) who studied the contributions of the British scientists Edwin S. Goodrich, Julian S. Huxley, and Gavin R. de Beer, and argued that morphology did, in fact, play an important role in the evolutionary synthesis, but that “all biologists had to use Darwinian interpretations of morphological findings in order to be considered as synthesists” in the first place (Waisbren 1988, p. 330).
